# Supplementary material for: Ran pathway-independent regulation of mitotic Golgi disassembly by Importin-α
Source: Nat Commun. 2019 Sep 20;10:4307. doi: 10.1038/s41467-019-12207-4 (PMC6754406; doi:10.1038/s41467-019-12207-4)
Supplement: Supplementary file 1 — Supplementary Information [file 41467_2019_12207_MOESM1_ESM.pdf]

Supplementary Information

Ran pathway-independent regulation of mitotic Golgi disassembly by Importin-α

Chih-Chia Chang[1], Ching-Jou Chen[2], Cédric Grauffel[3], Yu-Chung Pien[2], Carmay Lim[3], Su-Yi Tsai[2,4] & Kuo-Chiang Hsia[1,5]

[1]Institute of Molecular Biology, Academia Sinica, Taipei 11529, Taiwan.
[2]Department of Life Science, National Taiwan University, Taipei 10617, Taiwan.
[3]Institute of Biomedical Sciences, Academia Sinica, Taipei, Taiwan
[4]Genome and Systems Biology Degree Program, National Taiwan University and Academia Sinica, Taipei 10617, Taiwan
[5]Institute of Biochemistry and Molecular Biology, College of Life Sciences, National Yang-Ming University, Taipei 11221, Taiwan.

[*]Correspondence and requests for materials should be addressed to S.-Y. T. (e-mail: suyitsai@ntu.edu.tw), and K.-C. H. (e-mail: khsia@gate.sinica.edu.tw)

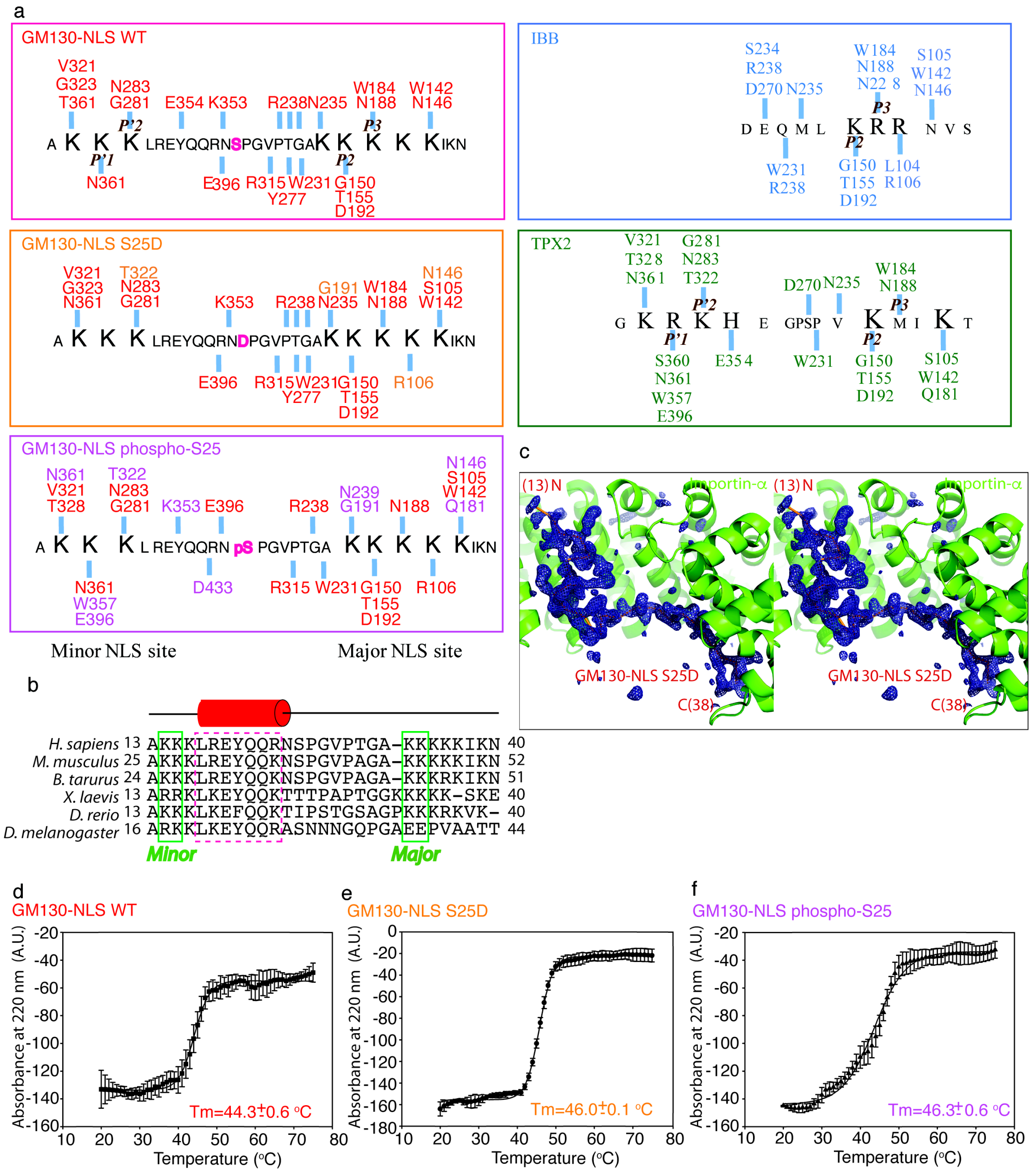

Supplementary Figure 1. The non-classical GM130-NLS and Importin-α interaction network. **a,** Schematic illustrations of the inter-molecular interaction network of Importin-α in complex with wild-type peptide, as well as peptides that contain a phosphomimetic residue or a phosphorylated moiety. Serine, aspartic acid and phosphoserine are highlighted in pink. Interaction networks of Importin-α with IBB and TPX2 are also shown. Blue lines indicate interaction residues. **b,** Sequence alignment of GM130-NLS peptides from different species (numbers represent amino acid positions). The conserved interacting residues of the minor binding site, central α-helix and major binding site are highlighted by boxes. The α-helix is represented as a red cylinder. **c,** Stereo view of an omit difference (Fo-Fc) map contoured at 2.5 sigma with a superimposed atomic model of the GM130-NLS S25D•Importin-α complex. GM130 and Importin-α are displayed as ribbons (orange) and cartoons (green), respectively. **d,e,f,** Circular dichroism spectra of temperature scans at a wavelength of 222 nm for the GM130-WT (**d**), S25D (**e**) or phospho-S25 (**f**) complexes. Each complex spectrum was the average of three scans. The error bars represent the standard deviation from three scans. Melting temperature (Tm) of each complex is indicated.

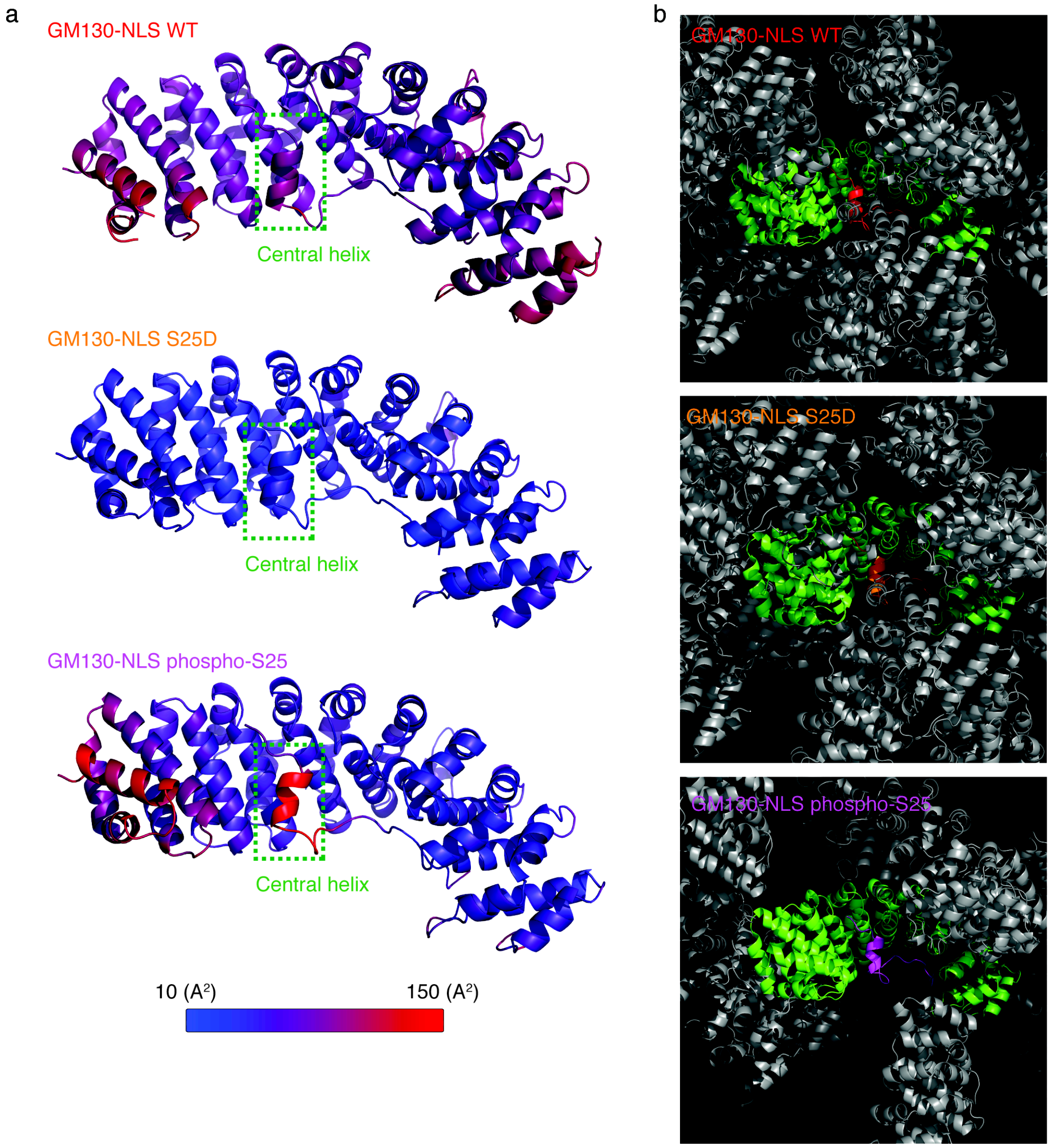

Supplementary Figure 2. B-factors of GM130•Importin-α complexes in different crystalline environments.

a, Structures of Importin-α (ΔIBB) in complex with GM130-NLS WT (top), GM130-S25D (middle) and phospho-S25 (bottom) are colored according to B-factor values, ranging from 10 $Å^2$ to 150 $Å^2$. The central helix is boxed by a green dashed line. **b,** Crystalline environments of Importin-α (ΔIBB) (green) in complex with GM130-NLS WT (top), GM130-S25D (middle) and phospho-S25 (bottom). Symmetry-related molecules are colored gray.

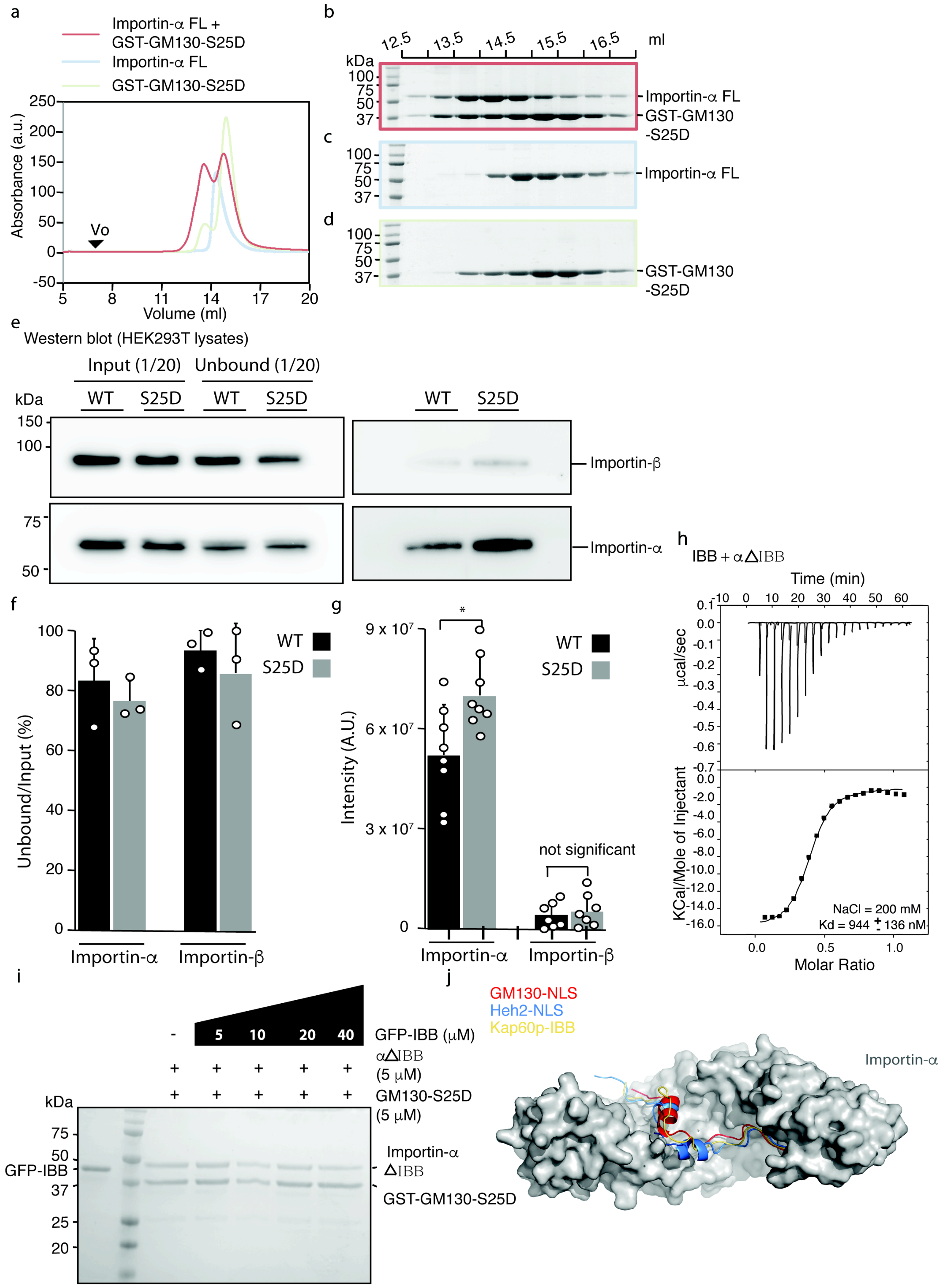
a
Importin-α FL +
GST-GM130-S25D
Importin-α FL
GST-GM130-S25D
Absorbance (a.u.)
Vo
Volume (ml)
b
12.5
13.5
14.5
15.5
16.5
ml
kDa
Importin-α FL
GST-GM130
-S25D
c
d
e
Western blot (HEK293T lysates)
Input (1/20)
Unbound (1/20)
WT
S25D
Importin-β
Importin-α
f
Unbound/Input (%)
g
Intensity (A.U.)
not significant
h
IBB + αΔIBB
Time (min)
μcal/sec
KCal/Mole of Injectant
NaCl = 200 mM
Kd = 944 ± 136 nM
Molar Ratio
i
GFP-IBB (μM)
αΔIBB
(5 μM)
GM130-S25D
(5 μM)
GFP-IBB
Importin-α
ΔIBB
GST-GM130-S25D
j
GM130-NLS
Heh2-NLS
Kap60p-IBB
Importin-α

Supplementary Figure 3. GM130 is resistant to IBB displacement upon interacting with Importin-α FL.

a-d, a, SEC (Superdex 200) elution profile for full-length Importin-α•GST-GM130-S25D (red line), full-length Importin-α (blue line), and GST-GM130-WT (green line). The peak fractions of full-length Importin-α•GST-GM130-S25D **(b)**, full-length Importin-α **(c)**, and GST-GM130-WT **(d)** were analyzed by SDS-PAGE and stained with Coomassie blue. The void volume (*Vo*) is indicated. **e,** Western blotting analysis of Importin-α pull-down by GST-GM130-WT and GST-GM130-S25D from HEK293T cell lysates. 1/20 of whole cell lysate (input) and of the total unbound proteins (unbound) is indicated. **f,g,** Band intensities of Importin-α and –β from the Western blotting in (**e**) were used to determine averages of protein in cell lysate **(f)**, unbound protein **(f)** and bound protein **(g)**. Data represent mean ± standard deviation from three independent experiments. Differences were assessed statistically by two-tailed Student's t test; $^*p$ <0.05. **h**, ITC titration curves (upper) and binding isotherms (lower) of Importin-α(ΔIBB) with IBB. The data was acquired under the same acquisition conditions as in Fig. 1D-F to determine Kd values for Importin-α(ΔIBB) with different GM130 peptides. Kd values are indicated. **i,** Competition binding assay. GST-GM130-S25D and Importin-α were incubated with indicated concentrations of GFP-IBB. Samples pulled down by GST beads were analyzed by SDS-PAGE and stained with Coomassie blue. **j**, Superimposition of the GM130-NLS peptide (red) with Heh2-NLS and Kap60p-IBB. Importin-α (70-498) is shown in gray.

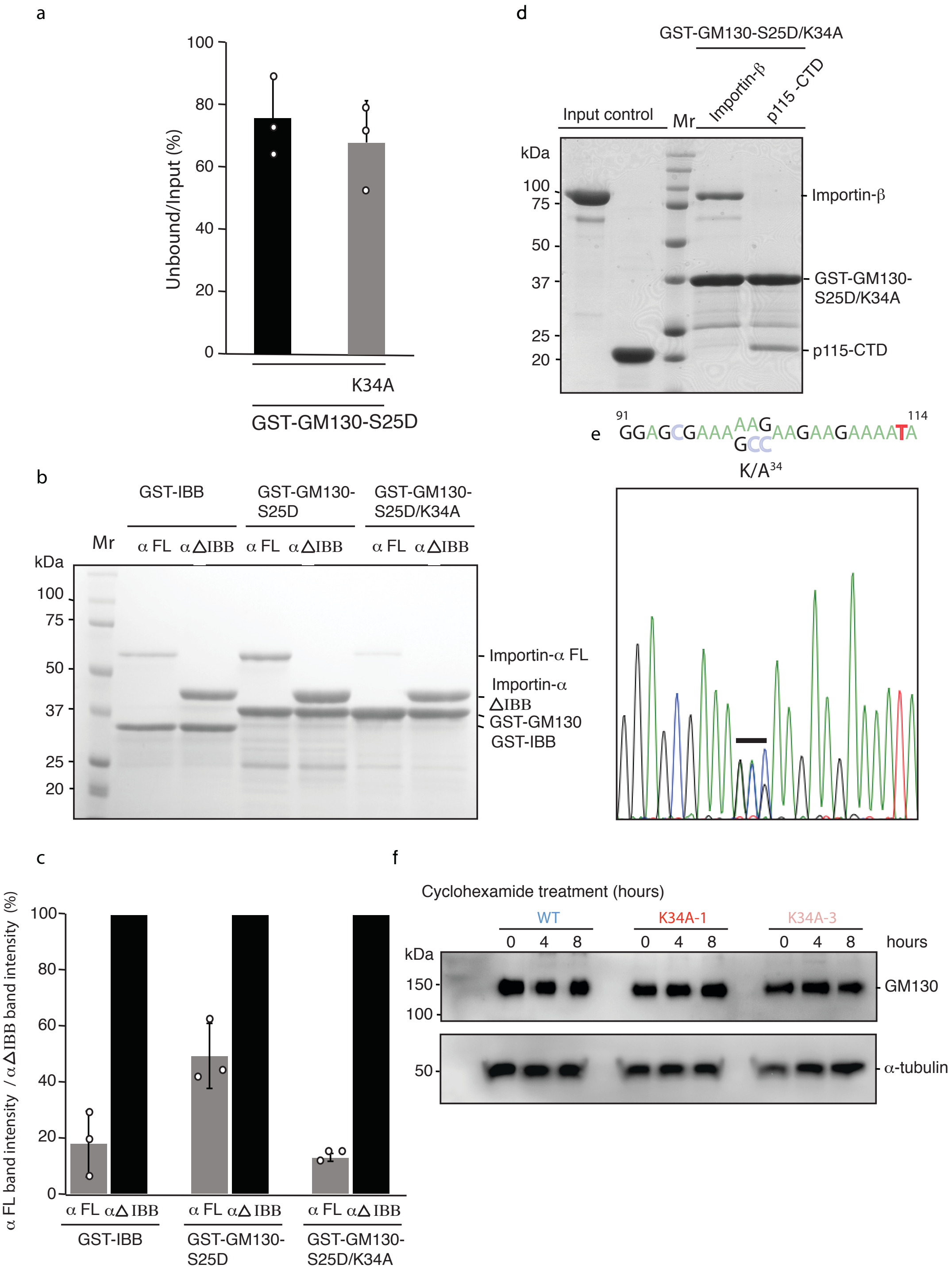
a
Unbound/Input (%)
100
80
60
40
20
0
K34A
GST-GM130-S25D
b
GST-IBB
GST-GM130-S25D
GST-GM130-S25D/K34A
Mr
α FL α△IBB α FL α△IBB α FL α△IBB
kDa
100
75
50
37
25
20
Importin-α FL
Importin-α △IBB
GST-GM130
GST-IBB
c
α FL band intensity / α△IBB band intensity (%)
α FL α△IBB
GST-IBB
GST-GM130-S25D
GST-GM130-S25D/K34A
d
GST-GM130-S25D/K34A
Input control
Mr
Importin-β
p115-CTD
kDa
GST-GM130-S25D/K34A
e
91
114
K/A34
f
Cyclohexamide treatment (hours)
WT
K34A-1
K34A-3
0 4 8
hours
150
100
GM130
50
α-tubulin

Supplementary Figure 4. Biochemical properties of GM130 and validation of cell lines containing the GM130 K34A mutation.

a, Band intensities of Importin-α from the Western blotting in **Fig. 5b** were used to determine averages of protein in cell lysate and unbound protein. Data represent mean ± standard deviation from three independent experiments. **b,** GST pull-down analyses of GST-tagged IBB, GM130-S25D or GM130-S25D/K34A incubated with 2-fold molar excess of either Importin-α FL or Importin-α (ΔIBB). **c,** Quantification of band intensities of bound Importin-α FL and Importin-α (ΔIBB) from the gel in (**c**), plotted as the ratio of Importin-α (ΔIBB) over Importin-α FL. Data represent mean ± standard deviation from three independent experiments. **d,** GST pulldown assays of GM130-S25D/K34A with Importin-β or p115-CTD. GST-fused GM130-S25D/K34A was incubated with Importin-β or p115-CTD at a molar ratio of 1:1. GST-bound samples were analyzed by SDS-PAGE and stained with Coomassie blue. **e,** A representative sequence electropherogram of the heterozygous GM130 K34A mutant. The black bar indicates the heterozygous nucleotides. Nucleotide and residue numbers are indicated. **f**, GM130 wild-type and K34A mutant HEK293T cell lines were mock-treated or treated with cycloheximide for the indicated timeframes, followed by Western blot analysis using GM130 and α-tubulin antibodies.

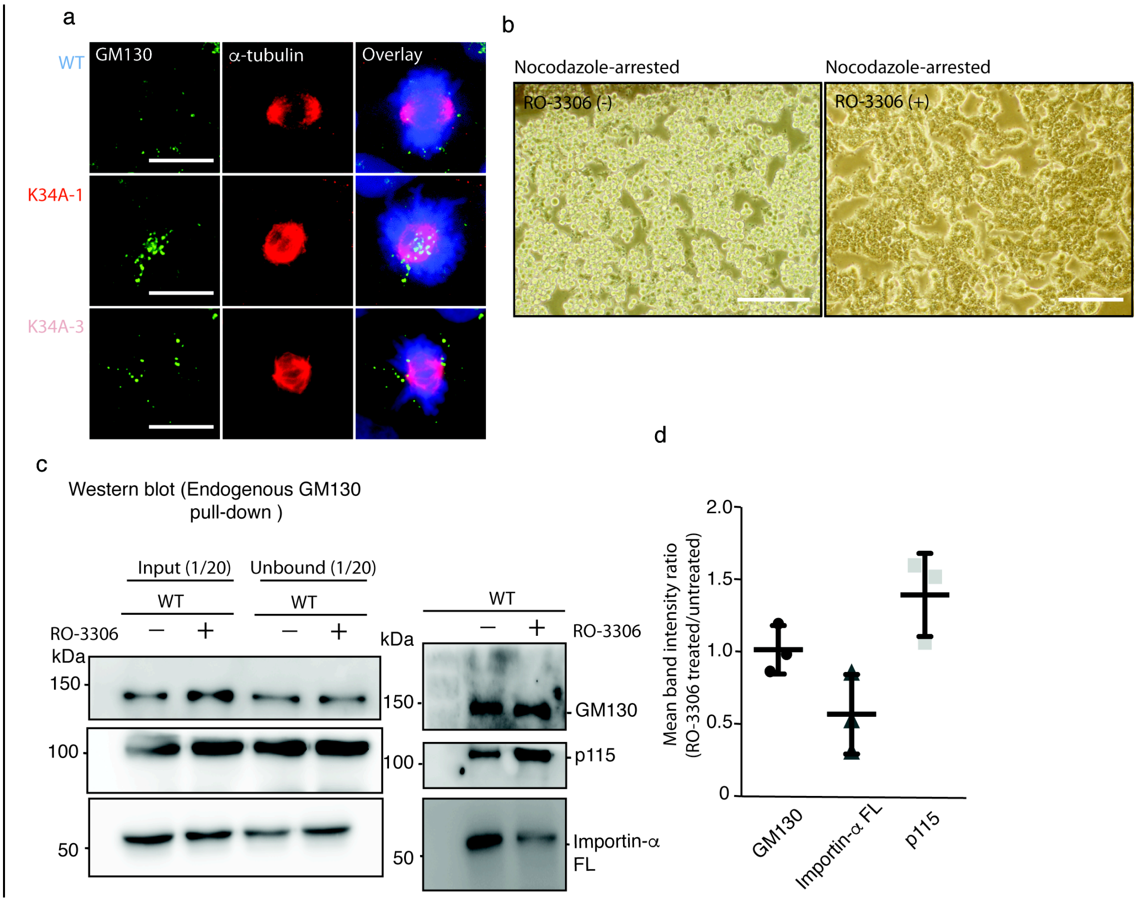

Supplementary Figure 5. Cell-based examination of the K34A mutation of GM130.

a, Representative fluorescent images of wild-type and two K34A mutant cell lines at metaphase. Cells were immuno-stained with GM130 antibody (green; left panel), α-tubulin (red, middle panel) and DAPI (blue, right panel). Scale bar: 10 µm. **b,** Images of nocodazole-arrested HEK293T cells without (left) and with (right) RO-3306 treatment acquired by bright-field illumination. Scale bar: 100 µm. **c,** Endogenous Importin-α and p115 were pulled down from nocodazole-arrested HEK293T cells treated (+) and untreated (-) with RO-3306 using GM130 antibody. Pulldown samples were analyzed by Western blot using antibodies for GM130, Importin-α and p115. 1/20 of whole cell lysate (input) and of the total unbound proteins (unbound) is indicated. **d,** Analysis of Importin-α and p115 pulldown by GM130 from RO-3306-treated and -untreated RO-3306 cell lysates. The scatter plot shows the ratios of the GM130, Importin-α and p115 band intensities for RO-3306 treated cells over untreated cells. Each dot represents an individual data point. Data represent mean ± standard deviation from three independent experiments.

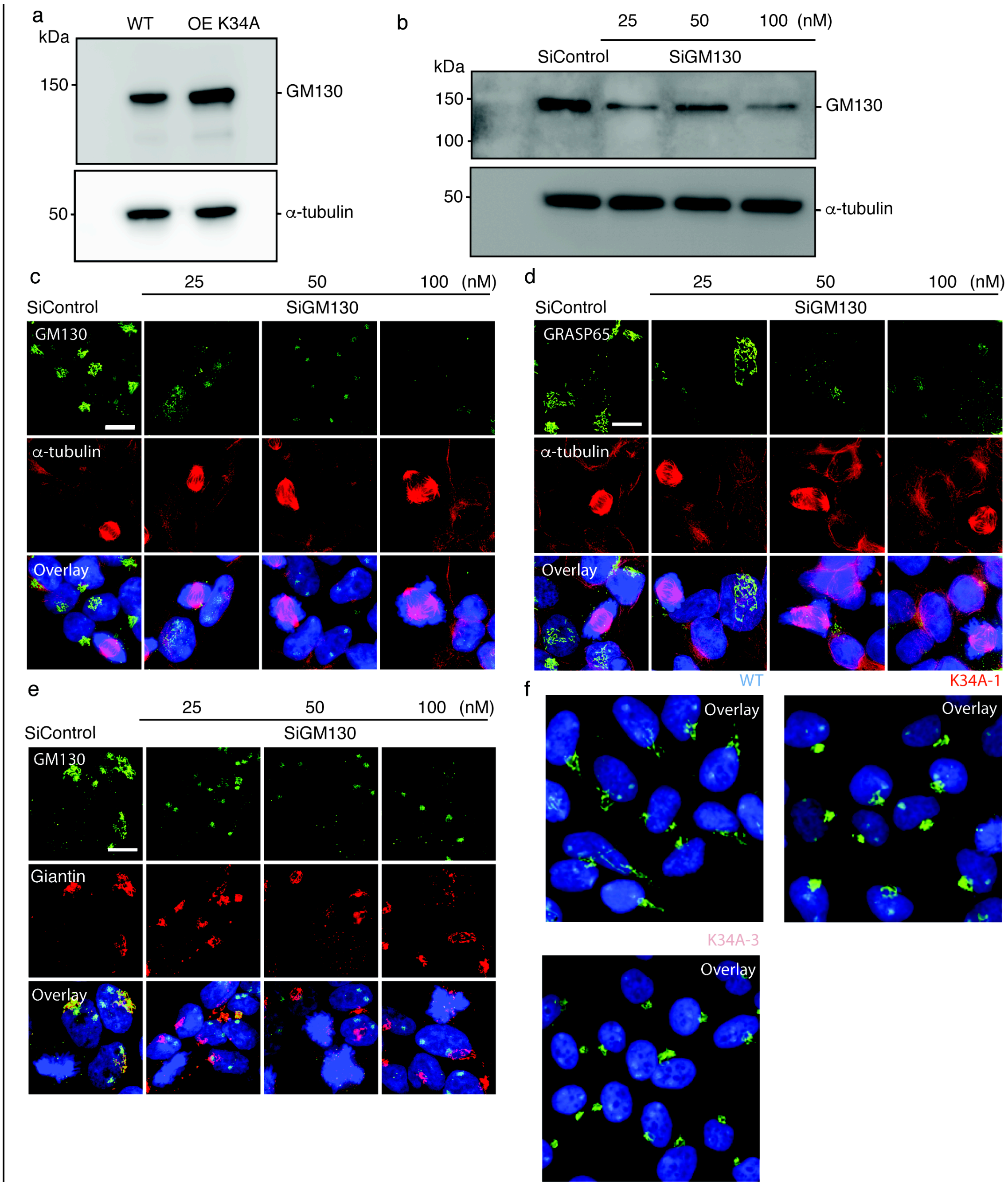

Supplementary Figure 6. Cell-based examination of GM130 K34A mutation overexpression and siRNA-mediated GM130 knockdown.

a, HEK293T cells overexpressing the GM130 K34A mutant were analyzed by Western blot using GM130 and α-tubulin antibodies. **b**, HEK293T cells were treated with indicated concentrations of siRNA oligonucleotides, followed by Western blot analysis using GM130 and α-tubulin antibodies. **c,d,e,** Representative fluorescent images of wild-type HEK293T cells treated with indicated siRNA oligonucleotides and subsequently immuno-stained with GM130 **(c)**, GRASP65 **(d)** or Giantin **(e)** antibodies. GM130 and GRASP65 are shown in green (top); α-tubulin and Giantin are shown in red (middle). Overlay images with DAPI staining (blue) are shown in the bottom panels. Scale bar: 10 μm. **f,** Merged immunofluorescence images of wild-type and K34A mutant cells in interphase. Cells were immuno-stained with GM130 antibody (colored green) and DAPI (colored blue).

Fig. 5b

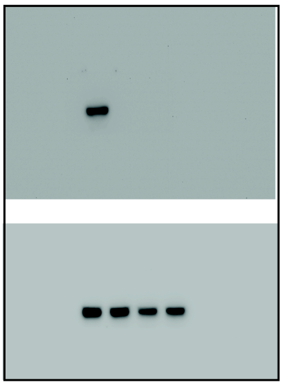

Fig. 6i

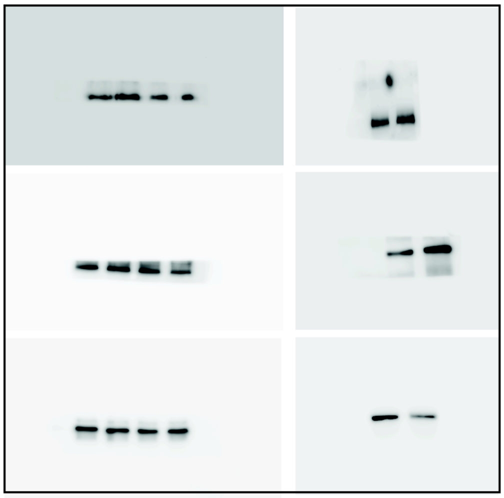

Supplementary Figure 3e

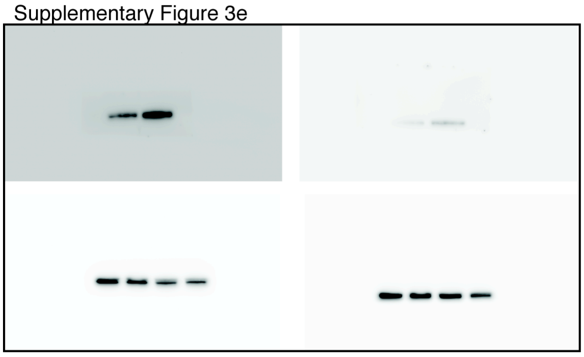

Supplementary Figure 4f

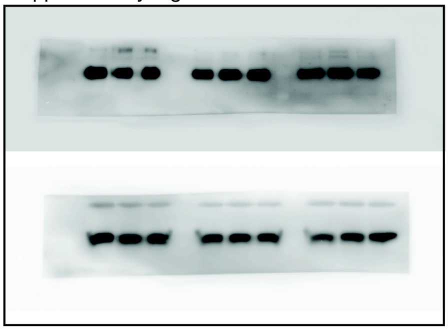

Supplementary Figure 5c

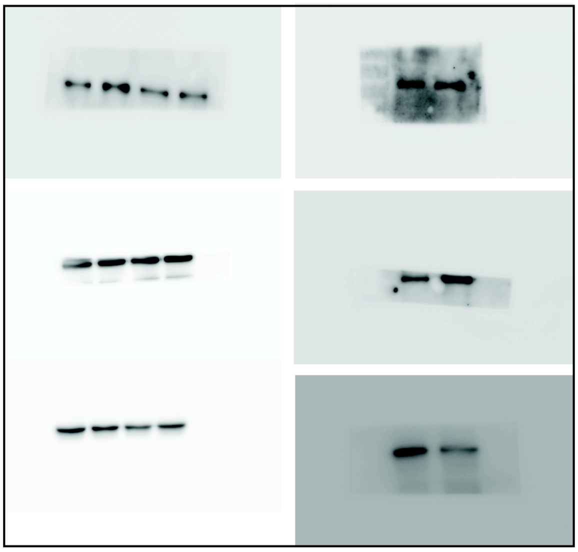

Supplementary Figure 6a

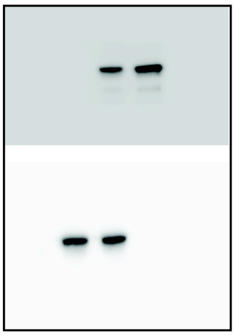

Supplementary Figure 6b

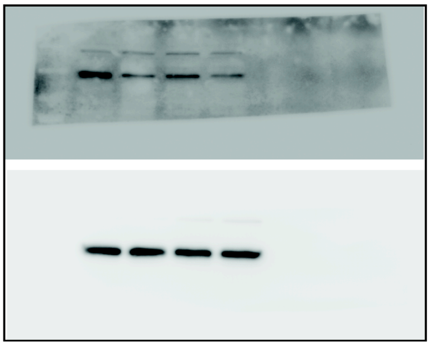

Supplementary Figure 7. Uncropped images of Western blots shown in the main and supplementary figures.

Supplementary Table 1: Primer sequences

**Primer**	**Sequence**
GM130 (1-48)-F	CTCCAAAATCGGATCCATGAGCGAAGAAACCCGTC
GM130 (1-48)-R	GGCCGCTCGAGTCGACTTAGGTGGTGGTTTCCGG
GM130 (1-85)-F	GGGGCCCCTGGGATCCATGAGCGAAGAAACCCGTC
GM130 (1-85)-R	GGCCGCTCGAGTCGACTTACAGGCTCGCGCCCGG
GM130 FL-F	CATAGAAGATTCTAGAATGTCGGAAGAAACCCGACAGA
GM130 FL-R	TCGCGGCCGCGGATCCTTAGATGACAGTGATCTTCACCTCA
GM130-S25D-F	TACCAGCAGCGTAAC GACCCGGGCGTTC
GM130-S25D-R	GAACGCCCGG GTC GTTACGCTGCTGGTA
GM130-K14A-F	AAACTGGCGGCGGCGGCGAAAAAACTGCGTGAATAC
GM130-K14A-R	GTATTCACGCAGTTTTTTCGCCGCCGCCGCCAGTTT
GM130-K34A-F	GTTCCGACCGGCGCGAAAGCGAAAAAGAAAATCAAAAAC
GM130-K34A-R	GTTTTTGATTTTCTTTTTCGCTTTCGCGCCGGTCGGAAC
p115 (780-930)-F	CGCGCGGCAGCCATATGGCTAGAGATTCTGAACAAGT
p115 (780-930)-R	GGTGGTGGTGCTCGAGTTAACCAAGATCCTTGAGTTT
Importin-α (70-478)-F1	CAGCCAGGATCCGAATTCGAACCAGGGTACTGTAAA
Importin-α (70-478)-R1	TTCTTTACCAGACTCGAGTTACACTGAGAAGTACTTCT
Importin-α (70-478)-F2	GGGGCCCCTGGGATCCAACCAGGGTACTGTAAATT
Importin-α (70-478)-R2	GGTGGTGGTGCTCGAGCACTGAGAAGTACTTCTCA
IBB-F	TTCAGGGCGCGGCCGCAATGTCCACGAACGAGAAT
IBB-R	CTCACCATGCCCTCGAG GTTCCGGTTTTCCTGTAG
Importin-α FL-F	AGGAGATATACCATGGGCATGTCCCCTATACTAGG
Importin-α FL-R	GGTGGTGGTGCTCGAGCACTGAGAAGTACTTCTCA
Importin-β FL-F	AGGAGATATACCATGGGCATGTCCCCTATACTAGG
Importin-β FL-R	GGTGGTGGTGCTCGAGTGCAGCTTCATAAGCAGCT
GM130 sgRNA-F	CACCGTTCTTCTTTTTCGCTCCTGT
GM130 sgRNA-R	AAAC ACAGGAGCGAAAAAGAAGAAC
GM130 Donor template	CTCATGAATATTATTGCTCTTCTTTTCCACAGTTGAGAGAATATC AGCAGAGGAATAGCCCTGGTGTTCCTACAGGAGCGAAAGCCAA GAAGAAAATAAAAAATGGCAGTAACCCTGAGACAACCACTTCT GGTGGTTGCCACTCACCTG
Genotype GM130-F	GCCATGGTATCAATCCCTCTCAG
Genotype GM130-R	TTTAGGACAGCAGGCCCTGTAC
