# Supplementary material for: Ran pathway-independent regulation of mitotic Golgi disassembly by Importin-α
Source: Nat Commun. 2019 Sep 20;10:4307. doi: 10.1038/s41467-019-12207-4 (PMC6754406; doi:10.1038/s41467-019-12207-4)
Supplement: Supplementary file 3 — Reporting Summary [file 41467_2019_12207_MOESM3_ESM.pdf]

nature research

Corresponding author(s): Kuo-Chiang Hsia

Last updated by author(s): Jul 19, 2019

Reporting Summary

Nature Research wishes to improve the reproducibility of the work that we publish. This form provides structure for consistency and transparency in reporting. For further information on Nature Research policies, see Authors & Referees and the Editorial Policy Checklist.

Statistics

For all statistical analyses, confirm that the following items are present in the figure legend, table legend, main text, or Methods section.

n/a	Confirmed	
☐	☒	The exact sample size (*n*) for each experimental group/condition, given as a discrete number and unit of measurement
☐	☒	A statement on whether measurements were taken from distinct samples or whether the same sample was measured repeatedly
☐	☒	The statistical test(s) used AND whether they are one- or two-sided<br>*Only common tests should be described solely by name; describe more complex techniques in the Methods section.*
☒	☐	A description of all covariates tested
☐	☒	A description of any assumptions or corrections, such as tests of normality and adjustment for multiple comparisons
☐	☒	A full description of the statistical parameters including central tendency (e.g. means) or other basic estimates (e.g. regression coefficient) AND variation (e.g. standard deviation) or associated estimates of uncertainty (e.g. confidence intervals)
☐	☒	For null hypothesis testing, the test statistic (e.g. F, t, r) with confidence intervals, effect sizes, degrees of freedom and P value noted<br>*Give P values as exact values whenever suitable.*
☒	☐	For Bayesian analysis, information on the choice of priors and Markov chain Monte Carlo settings
☐	☒	For hierarchical and complex designs, identification of the appropriate level for tests and full reporting of outcomes
☐	☒	Estimates of effect sizes (e.g. Cohen's *d*, Pearson's *r*), indicating how they were calculated

Our web collection on statistics for biologists contains articles on many of the points above.

Software and code

Policy information about availability of computer code

Data collection	*Provide a description of all commercial, open source and custom code used to collect the data in this study, specifying the version used OR state that no software was used.*
Data analysis	*Provide a description of all commercial, open source and custom code used to analyse the data in this study, specifying the version used OR state that no software was used.*

For manuscripts utilizing custom algorithms or software that are central to the research but not yet described in published literature, software must be made available to editors/reviewers. We strongly encourage code deposition in a community repository (e.g. GitHub). See the Nature Research guidelines for submitting code & software for further information.

Data

Policy information about availability of data

All manuscripts must include a data availability statement. This statement should provide the following information, where applicable:

- Accession codes, unique identifiers, or web links for publicly available datasets
- A list of figures that have associated raw data
- A description of any restrictions on data availability

All datasets presented in this manuscript are available from the corresponding author on request.

Field-specific reporting

Please select the one below that is the best fit for your research. If you are not sure, read the appropriate sections before making your selection.

☒ Life sciences ☐ Behavioural & social sciences ☐ Ecological, evolutionary & environmental sciences

Life sciences study design

All studies must disclose on these points even when the disclosure is negative.

Sample size	No sample size calculation were performed.
Data exclusions	No data were excluded from analyses.
Replication	All attempts at replication were successful. At least three independent replications were carried out for each qualification analysis.
Randomization	Samples were picked randomly to statistical analyses.
Blinding	The investigator assessing outcome will be blinded to treatment allocation.

Reporting for specific materials, systems and methods

We require information from authors about some types of materials, experimental systems and methods used in many studies. Here, indicate whether each material, system or method listed is relevant to your study. If you are not sure if a list item applies to your research, read the appropriate section before selecting a response.

Materials & experimental systems

n/a	Involved in the study	
☐	☒	Antibodies
☐	☒	Eukaryotic cell lines
☒	☐	Palaeontology
☒	☐	Animals and other organisms
☒	☐	Human research participants
☒	☐	Clinical data

Methods

n/a	Involved in the study	
☒	☐	ChIP-seq
☐	☒	Flow cytometry
☒	☐	MRI-based neuroimaging

Antibodies

Antibodies used

Immuno-precipitation: Anti-GM130(Abcam ab52649), Anti-importin-alpha(R&D Systems MAB6207), Anti-importin-beta(Abcam ab2811), Anti-p115(Abnova H00008615-M03), Anti-Rabbit-HRP (GeneTex GTX213110-01), Anti-Mouse-HRP (Jackson ImmunoResearch 115-035-003).

Immunofluorescence staining: Anti-GM130(Abcam ab52649), Anti-importin-alpha (R&D Systems MAB6207), Anti-GRASP65(Abcam-ab174834), Anti-giantin(Abcam-ab37266), Anti-alpha-Tubulin(GeneTex GXT628802), Anti-rabbit-Alexa Fluor 488 (Invitrogen A-21206), Anti-mouse-Alexa Fluor 561 (Invitrogen A-10037), ProLong Gold Antifade Mountant with DAPI (Invitrogen P36935).

Proximity ligation assay: Anti-GM130(Abcam ab52649), Anti-p115(Abnova H00008615-M03), ProLong Gold Antifade Mountant with DAPI (Invitrogen P36935), Duolink® In Situ PLA® Probe Anti-Mouse PLUS Affinity purified Donkey anti-Mouse IgG (Sigma DUO92001), Duolink® In Situ PLA® Probe Anti-Rabbit MINUS Affinity purified Donkey anti-Rabbit IgG (Sigma DUO92005)

Validation

Immuno-precipitation: Anti-GM130(rabbit), Anti-importin-alpha(mouse), Anti-importin-beta(mouse), Anti-p115(mouse), Anti-Rabbit-HRP (goat), Anti-Mouse-HRP (goat)

Immunofluorescence staining: Anti-GM130(rabbit), Anti-GRASP65(rabbit), Anti-giantin(mouse), Anti-importin-alpha (mouse), Anti-alpha-Tubulin(mouse),Anti-rabbit-Alexa Fluor 488 (donkey), Anti-mouse-Alexa Fluor 561 (donkey)

Proximity ligation assay: Anti-GM130(rabbit), Anti-p115(mouse), Duolink® In Situ PLA® Probe Anti-Mouse PLUS Affinity purified Donkey anti-Mouse IgG (donkey), Duolink® In Situ PLA® Probe Anti-Rabbit MINUS Affinity purified Donkey anti-Rabbit IgG (donkey)

Validation of primary antibody:
1. GM130: Validated in WB, IP, IHC, Flow Cyt, ICC/IF and tested in Cow, Dog, Human, Monkey, African green monkey.
2. Importin beta: Validated in Flow Cyt, ChIP, IP, Inhibition Assay, WB, ICC/IF and tested in Mouse, Rat, Cow, Dog, Human, Pig, African green monkey, Syrian hamster.
3. Importin alpha: Validated in WB, ELISAs and tested in human Importin alpha 2/KPNA2 and no cross-reactivity with recombinant human KPNA1, A3, A4, A5, or B1 is observed.
4. p115: Validated in WB, IHC, IF, ELISA and tested in Human USO1.
5. giantin: Validated in IHC, ICC/IF and react with Rat, Human.
6. GRASP65: Validated in ICC/IF, Flow Cyt, IP, IHC-P, WB and reacts with Human but does not react with: Mouse, Rat.

Eukaryotic cell lines

Policy information about cell lines

Cell line source(s)	HEK293T cell (ATCC® CRL11268) was used in immuno-precipitation, GST-pull down assay, cell culture, CRISPR/Cas9 targeting strategy, immunofluorescence staining, and proximity ligation assay.
Authentication	HEK293T cell line was authenticated by morphology using microscope.
Mycoplasma contamination	All cell lines were negative by mycoplasma test.
Commonly misidentified lines (See ICLAC register)	*Name any commonly misidentified cell lines used in the study and provide a rationale for their use.*

Flow Cytometry

Plots

Confirm that:

☒ The axis labels state the marker and fluorochrome used (e.g. CD4-FITC).

☒ The axis scales are clearly visible. Include numbers along axes only for bottom left plot of group (a 'group' is an analysis of identical markers).

☒ All plots are contour plots with outliers or pseudocolor plots.

☒ A numerical value for number of cells or percentage (with statistics) is provided.

Methodology

Sample preparation	cells were dissociated and washed with 1XPBS one time and fixed with 70% ethanol overnight. Cells were centrifuged and washed with 1xPBS one time. Cells were then re-suspended in 200 µl of Muse cell cycle assay kit (MCH100106)
Instrument	10,000cells /sample were analyzed
Software	Muse Cell Analyzer (Merck, Millipore).
Cell population abundance	cells are distributed in different stages of cell cycle including G1, S, G2 and M phases
Gating strategy	Based on different stages of cell cycle contain different amount of DNA content.

☒ Tick this box to confirm that a figure exemplifying the gating strategy is provided in the Supplementary Information.
